# Hybrid Multi-Biorecognition Laser-Scribed Graphene Platform for Electrochemical Detection of Insulin Resistance Biomarkers

**DOI:** 10.3390/bios16100545

**Published:** 2026-09-29

**Authors:** Lavita Nuraviana Rizalputri, Saptami Suresh Shetty, Mario Soto Martinez, Eckaard Le Roux, Veerappan Mani, Khaled Nabil Salama

**Affiliations:** 1 Bioengineering Program, Biomedical Science Division, King Abdullah University of Science and Technology, Thuwal 23955, Saudi Arabia; lavita.rizalputri@kaust.edu.sa (L.N.R.); saptami.shetty@kaust.edu.sa (S.S.S.); mario.sotomartinez@kaust.edu.sa (M.S.M.); 2 Electrical and Computer Engineering Program, Computer, Electrical and Mathematical Science and Engineering Division, King Abdullah University of Science and Technology, Thuwal 23955, Saudi Arabia; eckaard.leroux@kaust.edu.sa; 3 Centre for Advanced Measurement Research & Health Translation, Department of Pure and Applied Chemistry, University of Strathclyde, Glasgow G1 1XL, UK; veerappan.mani@strath.ac.uk

**Keywords:** laser-scribed graphene, electrochemical biosensor, multi-biorecognition platform, insulin resistance, insulin aptasensor, enzymatic glucose sensing, adipokine immunosensing

## Abstract

Insulin resistance (IR) is a multifactorial metabolic condition that can precede type 2 diabetes, motivating integrated monitoring of complementary biomarkers associated with glucose–insulin homeostasis and adipose tissue dysfunction. Here, we present a hybrid multi-biorecognition laser-scribed graphene (LSG) electrochemical platform with four independently functionalized working electrodes for detecting glucose, insulin, adiponectin, and leptin. The platform combines glucose oxidase-mediated enzymatic sensing, a methylene-blue-labeled insulin aptamer, and antibody-based recognition for adiponectin and leptin. Each sensing channel showed concentration-dependent electrochemical responses in PBS and analyte-spiked 10% human serum. The glucose sensor retained comparable sensitivity in PBS and serum, while the insulin aptasensor and the adiponectin and leptin immunosensors remained responsive in the diluted biological matrix. A preliminary mixed-analyte study further showed that all four channels remained responsive in a common four-biomarker sample. Importantly, the four-analyte panel combines glucose and insulin, used in HOMA-IR, with adiponectin and leptin, which form the adiponectin-to-leptin ratio, demonstrating thcombines high surface area, good electrical conductivitye feasibility of integrating complementary biomarkers and distinct biosensing approaches side by side within a single multiplexed electrochemical platform, with potential for future application in early-stage insulin resistance assessment.

## 1. Introduction

Insulin resistance (IR) is a metabolic condition in which major insulin-responsive tissues, including muscle, liver, and adipose tissue, become less responsive to normal insulin action [1,2]. Reduced insulin sensitivity initially triggers compensatory hyperinsulinemia, which can maintain near-normal glucose concentrations during the early stages of metabolic dysfunction [1,3]. Consequently, IR may develop before persistent hyperglycemia or overt clinical symptoms and commonly precedes type 2 diabetes mellitus [3]. Early identification of IR may therefore enable earlier risk stratification and intervention before progression to diabetes and related cardiometabolic complications [1,2,3,4], motivating the development of accessible approaches for monitoring biomarkers associated with IR.

The hyperinsulinemic-euglycemic glucose clamp (HEGC) is widely regarded as the reference method for assessing insulin sensitivity, but its complexity, cost, and requirement for trained personnel limit routine and point-of-care use [1]. Biomarker-based indices therefore provide more practical alternatives. The homeostasis model assessment of insulin resistance (HOMA-IR), derived from fasting glucose and insulin concentrations, is among the most established [1,5]. Beyond glucose–insulin homeostasis, IR is also closely linked to adipose tissue dysfunction, which contributes to obesity-related inflammation and metabolic impairment [6,7,8]. In this context, the adiponectin/leptin (A/L) ratio reflects the balance between insulin-sensitizing, anti-inflammatory adiponectin and adiposity-associated, pro-inflammatory leptin [6,8]. Lower A/L ratios are associated with impaired adipose tissue function and correlate with established measures of IR, including HOMA-IR, QUICKI, and HEGC [6,7,9,10,11]. Thus, glucose, insulin, adiponectin, and leptin provide complementary information related to glucose–insulin homeostasis and adipose tissue dysfunction.

In conventional workflows, these biomarkers are typically measured using separate analytical methods. Glucose is commonly measured using enzyme-based methods, whereas protein biomarkers such as insulin, adiponectin, and leptin are generally measured using immunoassay-based methods [12,13,14]. This separation increases workflow complexity, including sample handling, assay time, reagent consumption, and instrumentation requirements, whereas multiplexed or integrated biosensing approaches are specifically proposed to reduce sample volume, analysis time, and reagent cost by enabling multiplex biomarker detection [15,16]. However, such integration is technically challenging because the target analytes differ substantially in molecular properties and are best addressed using different biorecognition and signal-transduction strategies.

Electrochemical biosensing provides an attractive framework for this integration because of its sensitivity, compact instrumentation, and compatibility with miniaturized point-of-care formats [17,18]. Laser-scribed graphene (LSG) is particularly suitable for sensor-array fabrication because direct laser writing enables rapid patterning of porous, conductive graphene-like electrodes with high surface area and adaptable device geometries [19,20,21]. The resulting material combines a high surface area, good electrical conductivity, direct patternability, and compatibility with flexible or miniaturized device architectures, making it attractive for electrochemical sensor-array development [21,22].

Here, we report a hybrid multi-biorecognition LSG electrochemical platform comprising four independently functionalized working electrodes for glucose, insulin, adiponectin, and leptin. Our objective is to establish an integrated sensing architecture that accommodates four key IR-associated biomarkers on a single multiplexed platform. This platform’s distinguishing feature is the co-integration of four complementary IR-associated biomarkers spanning glucose–insulin homeostasis and adipose tissue dysfunction, using independently optimized recognition and electrochemical transduction mechanisms within a common LSG architecture. Rather than applying a single recognition chemistry to all targets, the platform combines bioreceptors selected for their compatibility with the corresponding electrochemical transduction mechanism. For glucose, glucose oxidase (GOx) was used because its highly specific enzymatic oxidation of glucose can be directly coupled to electrochemical signal generation through a redox mediator, providing a well-established catalytic sensing strategy [23]. For insulin, a thiolated, methylene-blue-labeled aptamer was employed because target-induced changes in aptamer conformation or steric environment can modulate electron transfer from the tethered redox reporter [24,25,26]. In contrast, adiponectin and leptin were detected using antibodies, well-established recognition elements for protein biomarkers and compatible with interfacial immunosensing based on restricted ferri/ferrocyanide electron transfer after antigen binding [27,28]. Thus, the hybrid design enables each sensing channel to use a recognition and signal-transduction strategy suited to its assay format rather than imposing a single biorecognition modality across all analytes. Each sensing interface was evaluated independently in PBS and analyte-spiked 10% human serum, and a preliminary mixed-analyte study was performed to assess inter-channel compatibility. This work therefore demonstrates the feasibility of integrating heterogeneous biorecognition strategies side by side within a common LSG architecture as a foundation for future multi-biomarker analysis of insulin resistance.

## 2. Materials and Methods

### 2.1. Materials and Reagents

Chloroauric acid (HAuCl_4_), 2-propanol, hydrochloric acid (HCl), potassium chloride (KCl), 3-mercaptopropionic acid (MPA), 1-ethyl-3-(3-dimethylaminopropyl)carbodiimide (EDC), N-hydroxysuccinimide (NHS), bovine serum albumin (BSA), glucose, glucose oxidase from *Aspergillus niger*, tris(2-carboxyethyl)phosphine (TCEP), 6-mercapto-1-hexanol (MCH), glutaraldehyde, magnesium chloride (MgCl_2_), Nafion™, acetone, ascorbic acid, and uric acid were purchased from Sigma-Aldrich (St. Louis, MO, USA). Potassium ferrocyanide (K_4_[Fe(CN)_6_]) and potassium ferricyanide (K_3_[Fe(CN)_6_]) were purchased from Thermo Scientific Chemicals (Geel, Belgium). Human insulin and ferrocene were purchased from Sigma-Aldrich (Schnelldorf, Germany), while human serum was purchased from Sigma-Aldrich (Merck KGaA, Darmstadt, Germany). Ag/AgCl paint was purchased from ALS Japan (Tokyo, Japan). Phosphate-buffered saline (PBS) tablets containing 0.0027 M KCl and 0.137 M sodium chloride (NaCl) were purchased from Fisher Scientific (Waltham, MA, USA). Adiponectin antibody, leptin antibody, recombinant adiponectin protein, and recombinant leptin protein were purchased from Abcam (Cambridge, UK). Insulin aptamers with a thiolated 5′ end and a 3′ end conjugated with methylene blue (100 μM), serving as a redox probe, were sourced from Integrated DNA Technologies (IDT; Leuven, Belgium). Polyimide (PI) sheets (0.76 mm thickness, 145 × 145 mm, Cirlex® CL grade) were purchased from UTECH Products GmbH (Willich, Germany). Kapton polyimide tape (TekLine Gold Kapton tape) was purchased commercially. All aqueous solution studies employed ultrapure water obtained from a Milli-Q® integrated water purification system (Merck KGaA, Darmstadt, Germany; resistivity: 18.2 MΩ·cm at 25 °C). 

### 2.2. Instruments

Surface morphology was examined with a Quattro scanning electron microscopy (SEM) system (Thermo Fisher Scientific, Waltham, MA, USA). Raman characterization was carried out at room temperature on a LabRAM ARAMIS instrument (HORIBA Scientific, Kyoto, Japan) using 532 nm excitation. Electrochemical measurements were performed using a PalmSens4 potentiostat (serial no. PS41911045829; PalmSens BV, Houten, The Netherlands) in combination with a MUX8-R2 multiplexer (serial no. MUX8R21904005703; PalmSens BV, Houten, The Netherlands). Data acquisition and instrument control were performed using PSTrace software (version 5.6; PalmSens BV, Houten, The Netherlands).

### 2.3. Fabrication of Multiplexed Laser-Scribed Graphene Electrodes (LSGE)

**Figure 1:**
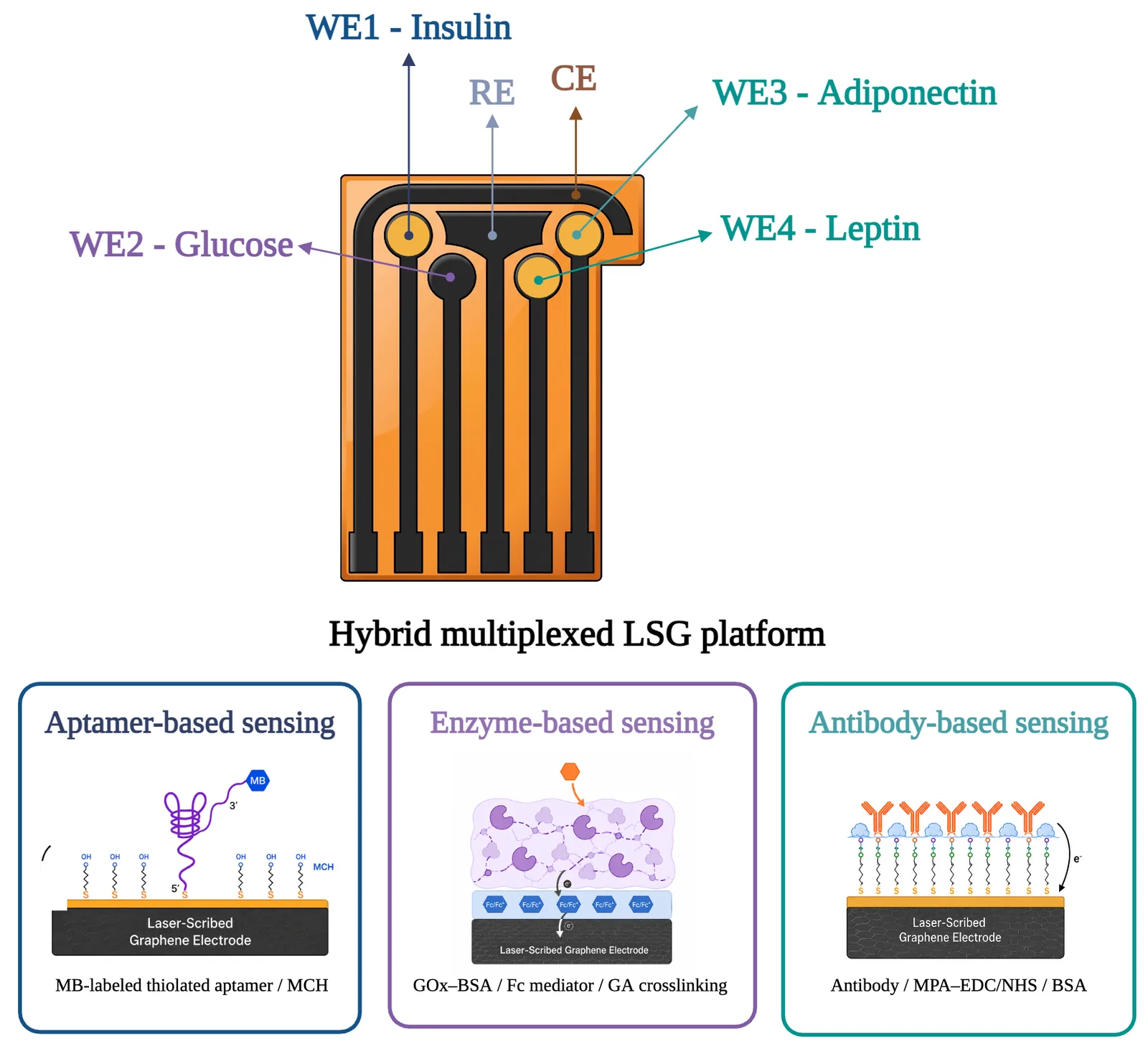
Schematic illustration of the hybrid multiplexed laser-scribed graphene (LSG) electrochemical platform integrating four working electrodes (WEs) for detection of insulin, glucose, adiponectin, and leptin. WE1 is configured for aptamer-based insulin sensing using a methylene blue (MB)-labeled thiolated aptamer immobilized on Au-modified LSG and passivated with 6-mercapto-1-hexanol (MCH). WE2 is configured for enzyme-based glucose sensing using glucose oxidase (GOx) and bovine serum albumin (BSA) immobilized by glutaraldehyde (GA) crosslinking above a ferrocene (Fc) redox-mediator layer. WE3 and WE4 are configured for antibody-based detection of adiponectin and leptin, respectively, using 3-mercaptopropionic acid (MPA), 1-ethyl-3-(3-dimethylaminopropyl)carbodiimide (EDC), and N-hydroxysuccinimide (NHS) chemistry for antibody immobilization, followed by BSA blocking. The four WEs share a common reference electrode (RE) and counter electrode (CE), enabling integration of aptamer-, enzyme-, and antibody-based biorecognition strategies within a single LSG platform.

LSGEs were prepared according to the fabrication procedure described in our earlier work [29]. The electrode layout was first created in AutoCAD 2025 (version V.58.M.214; Autodesk, Inc., San Francisco, CA, USA) and configured as an integrated electrochemical platform comprising a reference electrode (RE), a counter electrode (CE), and working electrodes (WEs). For this application, the design includes four separate WEs to detect different biomarkers (Figure 1). Before laser processing, the polyimide (PI) substrate was cleaned with 2-propanol and then irradiated with a CO_2_ laser (PLS4.75, 10.6 μm, 75 W; Universal Laser Systems, Inc., Scottsdale, AZ, USA) in a continuous nitrogen atmosphere to minimize heteroatom incorporation into the resulting graphene structure. Laser engraving was performed using the previously optimized conditions of 2.7 W laser power, 4.7 cm s^−1^ speed, 1000 pulses per inch, and a 2 mm *Z*-axis distance.

Following laser fabrication, Kapton tape was applied not only to electrically isolate the contact pads but also to precisely define the exposed sensing area of each working electrode. The same geometric opening was maintained across all four working electrodes to standardize the electrochemically accessible area and minimize variation from differences in solution exposure during Au electrodeposition, biofunctionalization, and electrochemical measurement. This additional geometric control improved electrode-to-electrode consistency across the multiplexed platform.

Three of the WEs from the multiplex LSGE design were decorated with gold nanostructures (LSGE/Au) fabricated by electrodeposition, following our previously established protocol with some modifications [30,31]. The deposition solution consisted of 50 mM HAuCl_4_ prepared in 0.5 M HCl, which served as the supporting electrolyte. A 100 µL aliquot of this solution was added to the electrochemical cell, after which chronoamperometric deposition was carried out at −0.90 V vs. the on-chip Ag/AgCl reference for 300 s. This treatment produced a visible, thin, yellow-colored gold layer on the electrode surface. After deposition, the modified electrodes were rinsed thoroughly with water at least three times to remove residual electrolyte prior to biofunctionalization. To prepare the reference electrode (RE), Ag/AgCl paint was applied to the designated reference-electrode region and then thermally cured in an oven at 90 °C for 10 min before electrochemical measurements.

### 2.4. Fabrication of Electrochemical Aptasensor for Insulin Detection

The insulin aptamer, originally described by Yoshida et al., was modified with a thiol at the 5′ end and methylene blue (MB) at the 3′ end (dual HPLC purification: 5′-SH-(CH_2_)_6_-AAA AGG TGG TGG GGG GGG TG GTA GGG TGT CT CT-methylene blue-3′) [31]. First, 2 µL of a 100 µM insulin aptamer solution was mixed with 14 µL of 10 mM TCEP for 1 h at room temperature (in the dark) to reduce disulfide species and generate/free the terminal thiol. Next, the solution was diluted to 1 µM in 1× PBS buffer containing 10 mM KCl and 2 mM MgCl_2_, heated to about 90 °C for 5 min, and then gradually cooled to about 4 °C. The annealing step promoted aptamer folding under conditions reported to favor G-quadruplex formation [24]. The aptamer was then diluted to 500 nM in the same buffer, and 10 µL of the solution was applied to the LSGE/Au working electrode 1 (WE1). The electrode was incubated for 2 h at room temperature in the dark and in a humid environment. After immobilization, the electrode was washed with 1× PBS and passivated with 10 µL of 10 mM MCH overnight to form a stable self-assembled monolayer. Finally, the modified electrodes were rinsed with 1× PBS and equilibrated in PBS for 2 h before analytical measurements (optimization later discussed in Section 3.2). For sample detection, 100 µL insulin in 1× PBS at different concentrations was applied to the working area and incubated for 3 minutes before electrochemical measurement. For serum measurements, human serum was diluted to 10% (*v*/*v*) in PBS and subsequently spiked with insulin at the specified concentrations. No additional pretreatment of the serum was performed. The endogenous concentrations of the target biomarkers in the commercial human serum were not independently quantified. Therefore, concentrations reported for serum-spiking experiments correspond to the added analyte concentrations. This also applied to other biomarkers (glucose, adiponectin, and leptin).

### 2.5. Fabrication of an Electrochemical Sensor with Enzyme for Glucose Detection

The glucose sensor was prepared by drop-casting 4 µL of a 0.1 M ferrocene solution containing 0.1% Nafion™ in acetone directly onto WE2 for 1 min, following a previously reported protocol with some modifications [23]. The small droplet remained confined to the WE2 area due to the hydrophobic character of the LSG surface, and no additional masking or physical isolation was used during this step. Then, the GOx solution (4 mg GOx and 7 mg BSA in 200 μL of 1× PBS) was mixed with 10 μL of 2.5% glutaraldehyde solution using a vortex mixer. A 10 μL aliquot of the mixture was drop-cast onto WE2 and dried at 4 °C for 24 h. For sample detection, glucose in 1× PBS at different concentrations was drop-cast onto the working area and measured immediately after deposition. For serum measurements, human serum was diluted to 10% (*v*/*v*) in PBS and subsequently spiked with glucose at the specified concentrations.

### 2.6. Fabrication of an Electrochemical Immunosensor for Adiponectin and Leptin Detection

WE3 and WE4 were functionalized using a modified version of our previously reported immunosensor protocol [30,31]. The Au-modified electrodes were exposed to 20 mM MPA for 1 h at room temperature to generate a carboxyl-terminated self-assembled monolayer. The surface carboxyl groups were then activated for 30 min using 4.0 µL of an EDC/NHS solution containing 50 mM of each reagent at a 1:1 molar ratio. Subsequently, 4.0 µL of the corresponding antibody solution (100 µg/mL in 1× PBS) was applied to the electrode and incubated for 16 h at 4 °C. The adiponectin-specific antibody was immobilized on WE3, whereas the leptin-specific antibody was assigned to WE4. To minimize nonspecific adsorption, each electrode was subsequently treated with 4.0 μL of BSA solution (0.1 mg/mL in 1× PBS) for 30 min. The electrode surfaces were rinsed with 1× PBS following each functionalization step. For target binding, 4.0 μL of the appropriate adiponectin or leptin solution was applied to the corresponding electrode and allowed to react for 30 min. In serum-based experiments, commercial human serum was first diluted to 10% (*v*/*v*) with PBS and then supplemented with the desired concentration of adiponectin or leptin. Electrochemical readout for both immunosensors was carried out in 5 mM ferro/ferricyanide prepared in KCl solution.

### 2.7. Electrochemical Sensing Measurement Parameters

The multiplexed device comprised four working electrodes (WEs) sharing a common reference and counter electrode (Figure 1). Three WEs were modified with LSGE/Au, while one used bare LSGE. The RE was an on-chip Ag/AgCl reference (LSGE painted with Ag/AgCl), and the counter electrode (CE) was made of LSGE. The chips were connected to the electrochemical workstation using an electrode connector provided by PalmSens. The cell volume was maintained at 100 µL.

CV, SWV, and chronoamperometry were performed for electrochemical characterization. The electrochemical studies were conducted in 1× PBS (pH 7.40) under ambient conditions unless otherwise stated in the text. For immunosensor measurements, SWV was performed over a potential range of −0.4 to +0.4 V with a potential step of 0.01 V, an amplitude of 0.1 V, and a frequency of 2.0 Hz. For the aptasensor, we used a potential range of −0.3 to 0.0 V with a potential step of 0.01 V, an amplitude of 0.1 V, and a frequency of 20.0 Hz (SWV frequency optimization is described in Section 3.2 and Figure S1C). For the enzyme measurement, chronoamperometry was conducted with a potential of 0.2 V and a time interval of 0.1 s for 100 s. Unless otherwise stated, all electrochemical measurements were performed at room temperature in triplicate.

Electrochemical signal changes were calculated relative to the corresponding matrix blank. For the signal-off insulin, adiponectin, and leptin sensors, ΔI was defined as ΔI = I_0_ − I_c_, where I_0_ is the peak current measured in the corresponding blank matrix (PBS or 10% human serum without added analyte), and I_c_ is the peak current measured after exposure to an added analyte concentration (C). For serum-spiking experiments, C represents the added analyte concentration because the endogenous concentrations of the target biomarkers in the commercial serum were not independently quantified. Unless otherwise stated, calibration data are reported as the mean ± standard deviation (SD) from *n* = 3 independently prepared electrodes.

The limit of detection (LOD) was estimated based on the variability of the blank response and the corresponding calibration relationship, following established analytical-validation principles [32,33]. For the linearly calibrated glucose sensor, the LOD was calculated as 3.3σ_blank_/S, where σ_blank_ is the SD of *n* = 3 independent blank measurements, and S is the calibration slope [33]. For sensors calibrated against a logarithmically transformed concentration, a ΔI_LOD_ = mean(ΔI_blank_) + 3.3σ_blank_ was first determined and subsequently converted to concentration using the inverse of the corresponding logarithmic calibration equation [32,33].

### 2.8. Combinatorial Analysis Preparation and Measurement

A preliminary combinatorial analysis was performed to evaluate the performance of the four sensing channels in a common mixed-analyte solution containing glucose, insulin, adiponectin, and leptin. For each target analyte, three concentrations were evaluated while the remaining biomarkers were maintained at fixed concentrations. The responses obtained in the mixed-analyte solution were compared with those obtained for the corresponding target analyte alone to assess the compatibility of the four sensing interfaces under mixed-sample conditions. For insulin, concentrations of 2.5, 10, and 100 µM were tested in the presence of 30 mM glucose, 100 µg/mL adiponectin, and 100 ng/mL leptin. For glucose, concentrations of 1, 10, and 30 mM were tested in the presence of 100 µM insulin, 100 µg/mL adiponectin, and 100 ng/mL leptin. For adiponectin, concentrations of 1, 10, and 100 µg/mL were tested in the presence of 100 ng/mL leptin, 100 µM insulin, and 30 mM glucose. For leptin, concentrations of 1, 10, and 100 ng/mL were tested in the presence of 100 µg/mL adiponectin, 100 µM insulin, and 30 mM glucose.

Because the four sensing interfaces use different electrochemical transduction mechanisms, the channels were measured sequentially rather than simultaneously. The adiponectin and leptin immunosensors were first prepared on WE3 and WE4, respectively, and their baseline currents were recorded using the ferri/ferrocyanide redox probe. The electrodes were subsequently rinsed and dried. The insulin aptasensor and glucose enzymatic sensor were then prepared on WE1 and WE2, respectively, followed by a 2 h PBS equilibration step for stabilization of the insulin aptamer interface. The insulin baseline response was recorded before addition of the mixed-analyte sample. After introducing 100 µL of mixed sample in 1× PBS, glucose was measured first by chronoamperometry for 100 s, followed immediately by SWV measurement of the insulin channel using the parameters described in Section 2.7. These measurements required approximately 5 min in total. The mixed sample was then maintained on the device for an additional 25 min, providing a total exposure time of approximately 30 min for the adiponectin and leptin immunosensors. The device was subsequently rinsed, and the adiponectin and leptin responses were measured in ferri/ferrocyanide solution as described in Section 2.7. Responses obtained in the mixed-analyte condition were compared with those obtained for the corresponding target analyte alone at the same concentration. All measurements were performed using *n* = 3 devices.

## 3. Results and Discussion

### 3.1. Bare LSGE and LSGE/Au Modification

**Figure 2:**
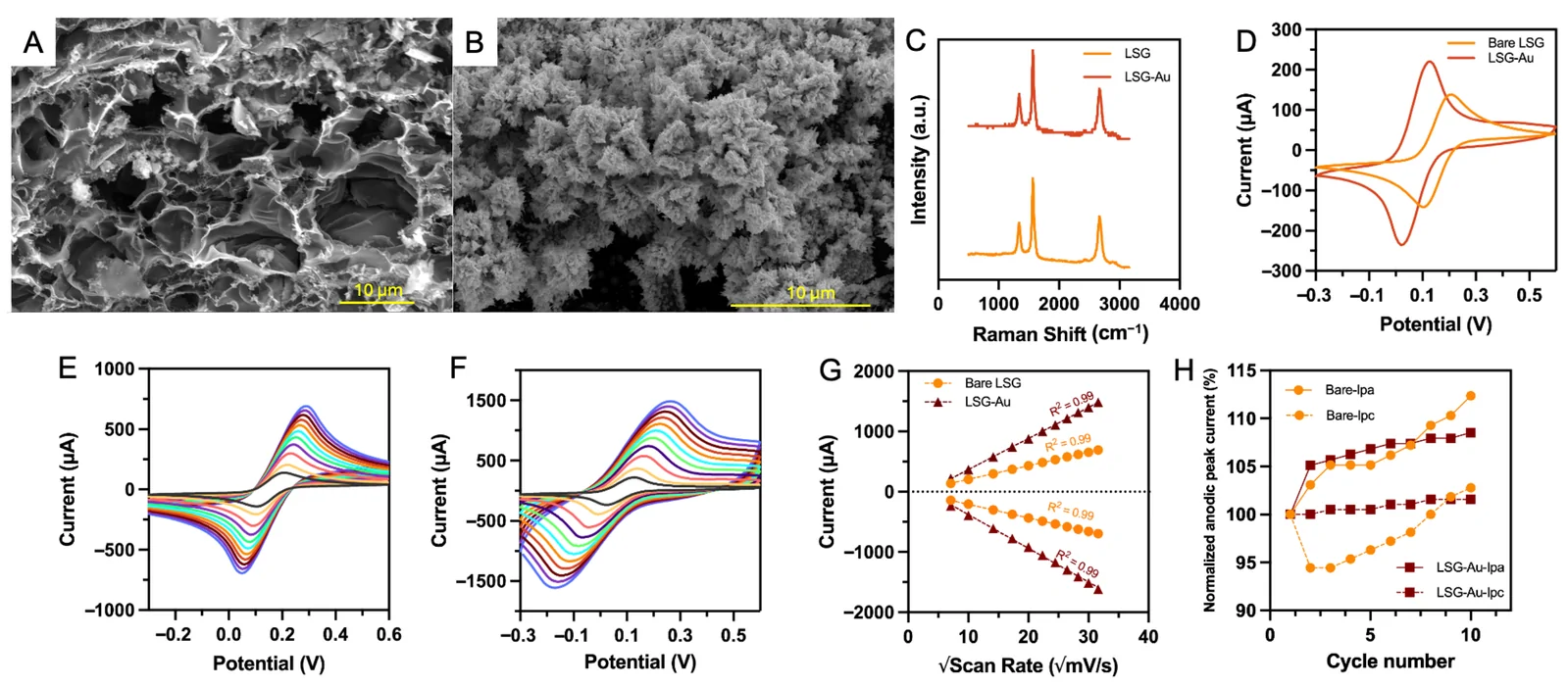
Characterization of LSGE and LSGE/Au. SEM of (**A**) LSGE and (**B**) LSGE/Au. (**C**) Raman spectra for the bare LSGE (orange) and LSGE/Au (red). (**D**) CVs before and after deposition of gold. CVs at scan rates from 0.1 to 1.0 V/s in increments of 0.1 V/s for (**E**) LSGE and (**F**) LSGE/Au. (**G**) Calibration data for different scan-rate measurements. (**H**) Short-term electrochemical cycling stability of bare LSGE and LSGE/Au. All measurements used 5.0 mM [Fe(CN)_6_]^3−/4−^ containing 0.1 M KCl.

Scanning electron microscopy (SEM) was used to examine changes in the surface morphology of the LSGE associated with Au deposition. Before imaging, the samples were coated with a 2 nm iridium layer to reduce charging during SEM analysis. Representative micrographs of the LSGE and LSGE/Au are shown in Figure 2A,B. The unmodified LSGE exhibited the expected porous, layered, sheet-like architecture (Figure 2A), characteristic of laser-scribed graphene electrodes and providing a high-surface-area framework for electrochemical sensing [27,30,34]. Following electrochemical deposition of gold on the working electrode, the LSGE surface became extensively covered with Au nanostructures (Figure 2B). Similar Au-decorated LSG morphologies have previously been reported by our group using electrochemical and other nanoparticle-deposition approaches [27,30,34,35].

Raman spectroscopy was subsequently employed to evaluate the structural characteristics and defect density of the graphene layers [27]. As illustrated in Figure 2C, both LSGE and LSGE/Au exhibited the characteristic D, G, and 2D Raman bands of graphene at approximately 1350, 1580, and 2685 cm^−1^, respectively, verifying the successful formation of graphitic structures on the PI substrate [27,36,37]. The calculated I_D_/I_G_ values were 0.85 for LSGE and 0.89 for LSGE/Au, indicating the degree of structural disorder in graphene [27,36,37]. The small difference between these values indicates that Au electrodeposition did not substantially change the defect level of the graphitic structure. Moreover, the comparable 2D-band intensities observed for LSGE and LSGE/Au suggest that the graphene layer number and overall electronic structure were largely preserved following gold deposition [37].

The electrochemical behavior of the unmodified and LSGE/Au electrodes is summarized in Figure 2D–G. The cyclic voltammograms presented in Figure 2D show higher oxidation and reduction peak currents for the LSGE/Au than for the bare electrode. This increase indicates a larger electrochemically active surface area together with more efficient electron-transfer kinetics [29]. These improvements likely stem from the deposited gold nanostructures, which introduce additional electroactive sites and promote charge transfer at the electrode–solution interface. In addition, the proportional dependence of peak current on the square root of scan rate, as shown in Figure 2E–G, is consistent with diffusion-controlled behavior. The greater slope obtained for the Au-modified electrode further demonstrates its enhanced electrochemical response relative to bare LSGE [29]. Thus, these findings show that incorporating Au nanostructures onto LSGE substantially improves electrochemical performance and will be advantageous for immobilizing biorecognition molecules, including aptamers and antibodies. The short-term electrochemical stability of bare LSGE and LSGE/Au was further evaluated over 10 consecutive CV cycles (Figure 2H). The anodic and cathodic peak currents were normalized to the corresponding first-cycle values. For bare LSGE, the normalized anodic current increased to approximately 112.4% by cycle 10, while the cathodic current initially decreased to approximately 94.4% before recovering to 102.8%. In comparison, LSGE/Au showed smaller overall changes, reaching approximately 108.5% and 101.6% for the anodic and cathodic peak currents, respectively. These results indicate modest signal evolution during repeated cycling, with comparatively smaller variation in the cathodic response of the Au-modified electrode.

### 3.2. Analytical Performance of the Insulin Aptasensor

Prior to sensor optimization, the insulin aptamer sequence and its predicted secondary structure were evaluated using UNAFold (https://www.unafold.org/; accessed on 13 August 2026), as shown in Figure S1A. The aptamer was designed with a thiol modification at the 5′ end to enable covalent immobilization on the Au-decorated laser-scribed graphene surface via Au–S bonding, and a methylene blue (MB) redox tag at the 3′ end to enable electrochemical signal transduction via SWV. This configuration anchors the aptamer to the sensor surface and positions the MB label as the electroactive reporter for monitoring aptamer-based binding events. The immobilization of the insulin aptamer on the Au-decorated laser-scribed graphene sensor was then optimized by varying the gold deposition time using a gold seed solution containing HAuCl_4_ in HCl. This optimization was performed because increasing the amount of deposited gold is expected to increase the available Au surface area for thiol–gold binding, thereby providing more anchoring sites for the thiol-modified aptamer [38]. As shown in Figure S1B, the SWV peak current of methylene blue increased with increasing gold deposition time from 60 to 300 s, rising from approximately 2.0 to 7.2 µA. This trend indicates that longer gold deposition enhances aptamer immobilization, consistent with forming a higher density of Au–thiol-linked aptamers on the sensor surface. However, the signal decreased to 6.2 µA at 360 s, possibly due to excessive Au growth. Similar behavior has been reported during Au nanoparticle electrodeposition, where longer deposition times increased particle growth and altered nanoparticle size/dispersion [27,39]. Therefore, excessive deposition may reduce the effectively accessible Au surface area and hinder electron transfer between the MB label and the electrode surface. Based on these results, 300 s was selected as the optimal gold deposition time.

The SWV frequency was then optimized to obtain the highest MB response from the immobilized insulin aptamer. As shown in Figure S1C, the MB peak current strongly depended on frequency, with the highest signal at 20 Hz. Increasing the frequency beyond this value progressively decreased the peak current, reaching a nearly stable low-current response at higher frequencies. This behavior indicates that the electron-transfer kinetics of the MB-labeled aptamer on the LSGE/Au surface are better resolved at lower SWV frequencies [40]. At higher frequencies, the measurement timescale may become too fast relative to the MB electron-transfer process and aptamer motion, resulting in incomplete redox conversion and lower peak current [40]. Therefore, 20 Hz was selected as the optimal SWV frequency for subsequent measurements. Finally, the post-fabrication equilibration time in PBS was investigated by monitoring the MB peak current after completion of aptamer immobilization and MCH passivation (Figure S1D). The MB peak current increased progressively during PBS incubation and approached a plateau after approximately 120 min. Therefore, a 2 h equilibration period was selected before analytical measurements. The time-dependent increase in MB current observed here is consistent with post-fabrication equilibration of the aptamer/MCH interface toward a more stable dynamic configuration before analytical measurements [41,42]. Post-fabrication conditioning of electrochemical aptamer-based sensors in PBS has similarly been used to stabilize the sensor interface following aptamer and MCH assembly [43].

**Figure 3:**
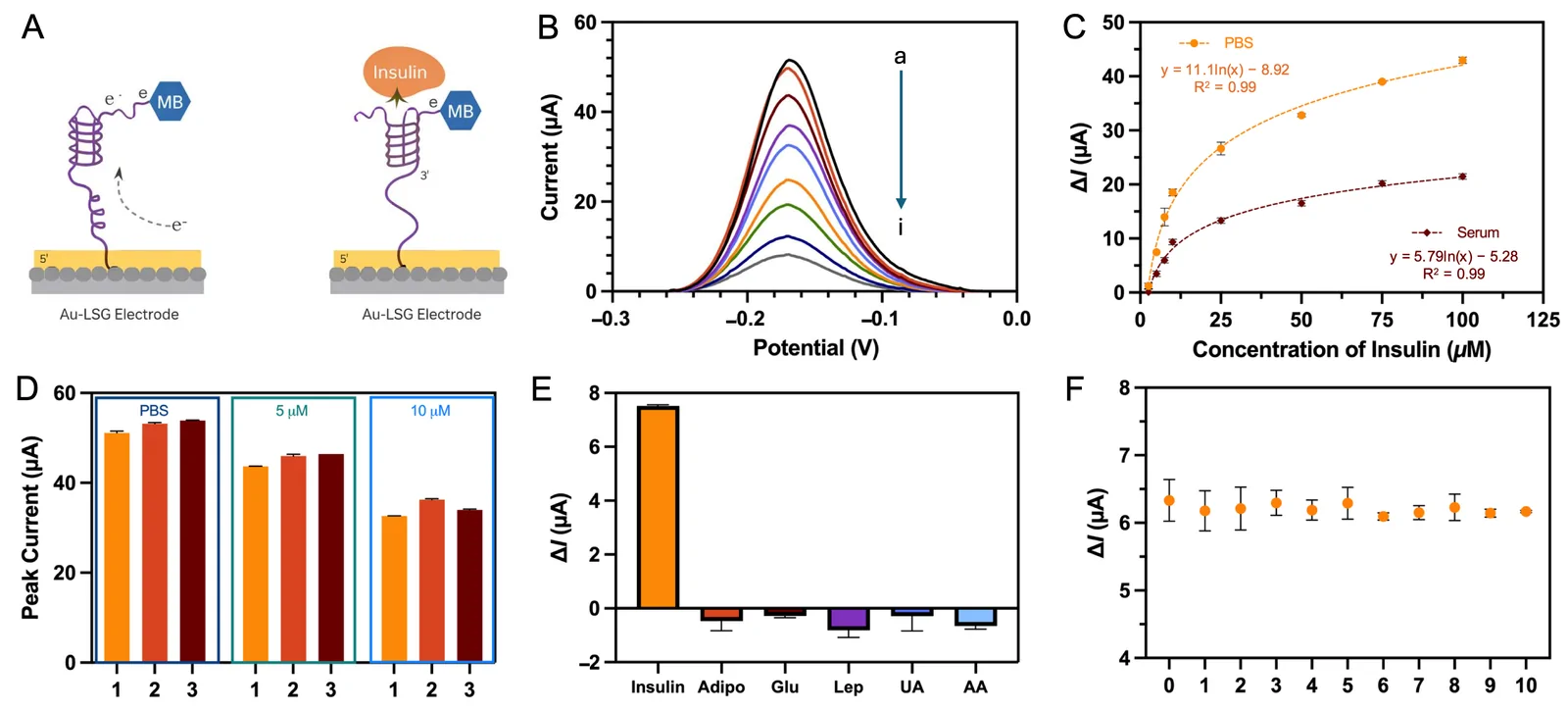
(**A**) Schematic illustration of the proposed sensing mechanism, in which insulin binding changes the configuration of the MB-labeled aptamer, reducing electron-transfer efficiency at the LSGE/Au electrode surface. (**B**) Square-wave voltammetry responses obtained at increasing insulin concentrations: (a) 0, (b) 2.5, (c) 5, (d) 7.5, (e) 10, (f) 25, (g) 50, (h) 75, and (i) 100 µM in PBS. (**C**) Calibration curve showing the relationship between the change in current (ΔI) and insulin concentration in PBS (orange) and human serum (purple) environments. (**D**) Reproducibility assessment of the sensor at different insulin concentrations (PBS, 5 µM, and 10 µM) across multiple electrodes. (**E**) Equimolar cross-reactivity assessment comparing the response toward insulin (5 µM) with adiponectin, glucose, leptin, uric acid (UA), and ascorbic acid (AA), each tested at 5 µM. (**F**) Repeatability evaluation of the sensor over repeated measurements, demonstrating a consistent signal response over multiple cycles. All measurements were repeated three times.

Using the optimized parameters, the electrochemical performance of the aptamer-functionalized LSGE/Au electrode for insulin detection was systematically evaluated using square-wave voltammetry (SWV). Figure 3A illustrates the proposed sensing mechanism of the MB-labeled insulin aptasensor. The observed decrease in MB peak current upon insulin binding is consistent with a target-induced change in the aptamer configuration and/or steric environment that reduces the electron-transfer efficiency of the distal MB redox reporter [44]. Similar signal-off behavior has been reported for electrochemical insulin aptasensors employing G-quadruplex-forming, redox-labeled aptamers, where target binding alters the accessibility and electron-transfer kinetics of the redox reporter [44].

This behavior is clearly reflected in the SWV responses (Figure 3B), with a concentration-dependent decrease in peak current as insulin levels increase. The corresponding calibration curve (Figure 3C) shows a logarithmic relationship (R^2^ = 0.99), typical of affinity-based biosensors due to progressive saturation of binding sites at higher analyte concentrations [45]. At lower concentrations, the response is driven by high-affinity binding events, whereas at higher concentrations, the signal tends to plateau as the aptamer sites become increasingly occupied [25,46]. Measurements were also performed in serum (Figure S2A). A similar concentration-dependent trend (R^2^ = 0.99) was observed in analyte-spiked 10% human serum (Figure 3C), although the peak currents and overall signal change are lower and the voltammetric profiles are slightly broader, which may arise from matrix effects associated with serum proteins and other endogenous components [27]. In PBS, the response follows ΔI = 11.1 ln(C) − 8.92, whereas in serum, the response follows ΔI = 5.79 ln(C) − 5.28. Thus, the slope obtained in serum is approximately 52% of that observed in PBS, indicating reduced analytical sensitivity in the complex biological matrix. This attenuation may be attributed to nonspecific adsorption, partial surface blocking by serum constituents, and differences in the interfacial environment that can hinder target diffusion or electron transfer [47,48,49]. Nevertheless, the logarithmic dependence on concentration and high linearity in human serum demonstrate that the sensing platform retains a concentration-dependent insulin response within the investigated concentration range. The calculated LODs were 2.29 µM in PBS and 2.71 µM in 10% human serum. The insulin concentration range investigated in the present study is higher than that required for direct analysis of fasting physiological insulin levels [50]. Therefore, the present aptasensor should be regarded as a proof-of-concept insulin-responsive sensing interface.

The sensitivity limitation may not be intrinsic to the aptamer recognition principle itself, as Nandhakumar et al. demonstrated picomolar insulin detection using a methylene-blue-labeled G-quadruplex aptamer [24]. This suggests that the higher LOD obtained in this LSG-based platform work may arise, at least in part, from differences in electrode and interfacial architecture. In electrochemical aptamer sensors, probe packing density, surface chemistry, and reporter–electrode distance can strongly influence target accessibility, conformational freedom, and signal gain [26,51,52]. Although the porous Au-modified LSG provides a high electroactive surface area and supports aptamer immobilization, a high absolute MB current does not necessarily correspond to an optimal target-induced response. Variations in local probe density and aptamer accessibility across the heterogeneous LSG/Au surface may therefore contribute to the reduced insulin sensitivity. Future work should focus on controlling Au morphology, quantifying aptamer surface density, and optimizing the aptamer/MCH interface to improve coupling between insulin binding and MB electron transfer. Alternative redox reporters such as ferrocene could also be evaluated to determine whether different electron-transfer characteristics improve signal gain. If further sensitivity enhancement is required, additional amplification approaches, including catalytic amplification, redox cycling, or nucleic-acid-based target recycling, could also be incorporated [53,54].

The reproducibility of the sensing platform was assessed using three independently prepared electrodes in PBS, 5 µM insulin, and 10 µM insulin (Figure 3D). For each concentration, the three electrodes produced closely comparable peak-current values, with only minor variation among replicates. In PBS, the measured currents remained within a narrow range of approximately 50–53 µA. Similarly, at 5 µM insulin, the peak currents were around 44–46 µA, while at 10 µM insulin they were approximately 32–36 µA. The small error bars and limited electrode-to-electrode variation demonstrate good fabrication reproducibility and consistent electrochemical performance. An initial cross-reactivity assessment was performed by comparing the response toward insulin (5 µM) with those obtained for individual non-target species at the same nominal concentration (Figure 3E). Under these equimolar test conditions, insulin produced a substantially larger response than adiponectin, glucose, leptin, uric acid (UA), and ascorbic acid (AA), indicating preferential recognition of the target by the aptamer interface. These experiments were designed as controlled specificity tests rather than to reproduce physiological analyte-to-interferent concentration ratios. Because electrochemical biosensors may encounter interfering serum components at concentrations substantially higher than those of low-abundance protein biomarkers, future studies should evaluate interference using physiologically representative concentration ratios and clinically relevant matrices [55]. The operational repeatability of the sensor was evaluated over 11 consecutive measurements, as shown in Figure 3F. The ΔI values remained highly consistent throughout the testing sequence, fluctuating only slightly around approximately 6.1–6.3 µA. The relatively small error bars further demonstrate good measurement precision and limited variability between replicates. Overall, these results indicate that the sensing platform exhibits good short-term signal repeatability, with minimal signal drift during repeated electrochemical measurements.

### 3.3. Analytical Performance of the Glucose Enzymatic Sensor

**Figure 4:**
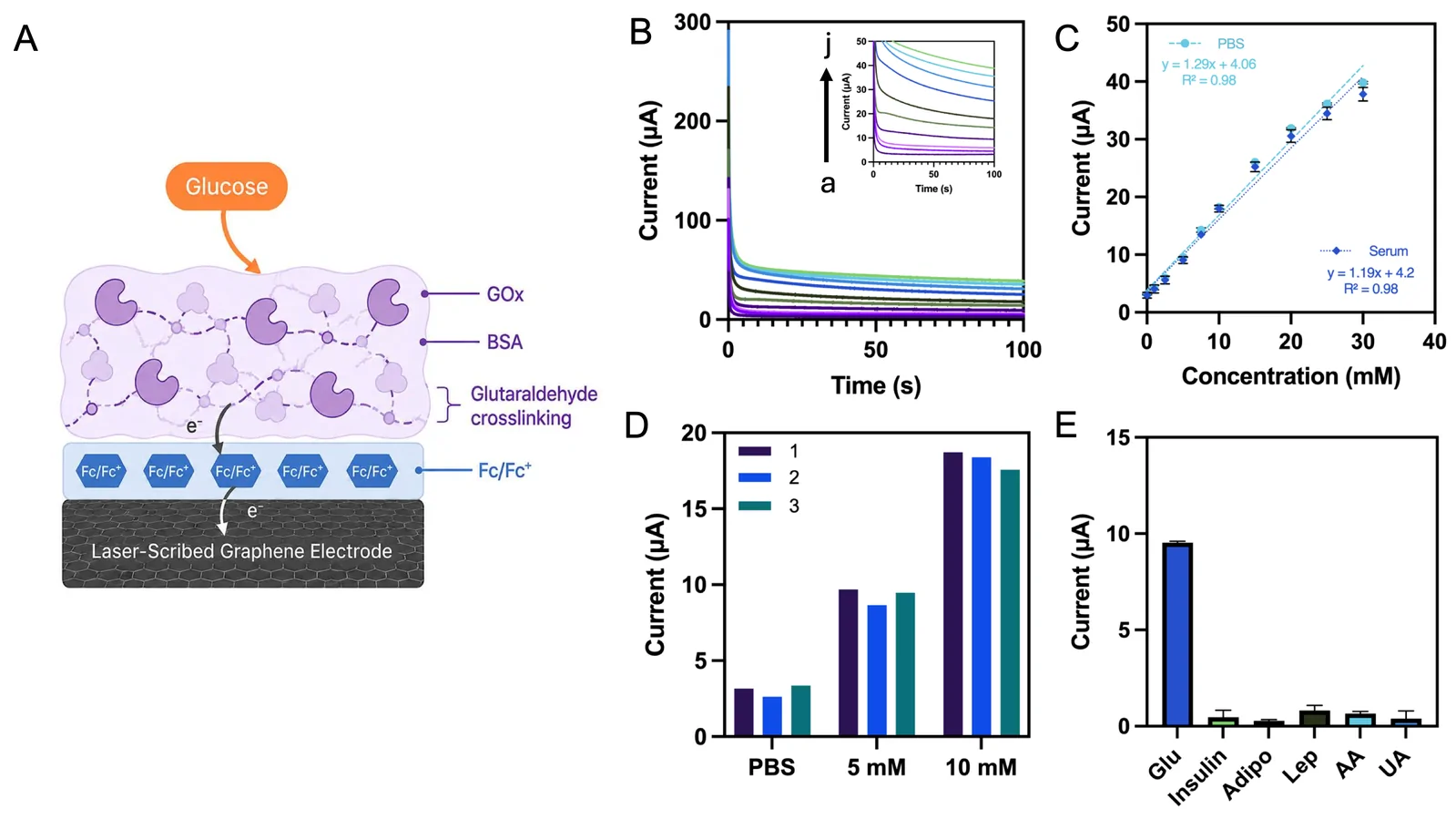
Electrochemical detection of glucose using a GOx-functionalized LSGE. (**A**) Schematic illustration of the proposed ferrocene-mediated enzymatic glucose-sensing mechanism. (**B**) Chronoamperometric responses obtained at increasing glucose concentrations: (a) 0, (b) 1, (c) 2.5, (d) 5, (e) 7.5, (f) 10, (g) 15, (h) 20, (i) 25, and (j) 30 mM in PBS (inset: enlarged view of current response over time). (**C**) Calibration curve demonstrating the linear relationship between current and glucose concentration in PBS and human serum. (**D**) Reproducibility evaluation of the sensor at different concentrations (PBS, 5 mM, and 10 mM) across independently fabricated electrodes. (**E**) Equimolar cross-reactivity assessment comparing the response toward glucose (5 mM) with insulin, adiponectin, leptin, ascorbic acid (AA), and uric acid (UA), each tested at 5 mM.

Figure 4A illustrates the working principle of the mediated enzymatic glucose sensor, in which glucose oxidase (GOx) is immobilized together with BSA through glutaraldehyde crosslinking above a ferrocene/ferrocenium (Fc/Fc^+^) mediator layer supported on the laser-scribed graphene electrode. Upon exposure to glucose, GOx catalyzes its oxidation, thereby reducing the enzyme-bound FAD cofactor to FADH_2_ [23]. The reduced enzyme then transfers electrons to the oxidized mediator, ferricenium (Fc^+^), thereby regenerating GOx-FAD and reducing Fc^+^ to ferrocene (Fc) [23]. Ferrocene is then electrochemically oxidized back to Fc^+^ at the LSGE, producing the measured faradaic current and regenerating the mediator for continued electron transfer [23]. In Figure 4B, the chronoamperometric response shows an initially high current that rapidly decreases before reaching a quasi-steady-state value, consistent with the decay of transient charging effects and establishment of a stable faradaic response [56]. The steady-state current then increased with glucose concentration, confirming a concentration-dependent sensor response, as observed in other enzymatic chronoamperometric glucose sensors [23,57]. The mean current over the final 30 s of each 100 s measurement was calculated and plotted in Figure 4C. A linear increase in current with glucose concentration was observed in both PBS and analyte-spiked 10% human serum, with similar sensitivities of 1.29 and 1.19 µA mM^−1^, respectively (R^2^ = 0.98). The relatively small difference indicates good tolerance of the glucose sensor to the serum matrix under the tested conditions. This behavior may be associated with the GOx-mediated catalytic reaction and Fc/Fc^+^-assisted electron-transfer pathway, which can support efficient signal generation despite moderate matrix interference [56,58]. In contrast, the insulin aptasensor relies more directly on target-induced changes at the electrode interface and is therefore more susceptible to nonspecific adsorption and interfacial blocking by serum components [59,60]. The calculated LODs for glucose were 0.57 mM in PBS and 1.06 mM in human serum. The investigated glucose range (1–30 mM) encompasses clinically relevant circulating glucose concentrations, including fasting plasma glucose levels associated with impaired fasting glucose (5.6–6.9 mM) and diabetes (≥7.0 mM), and the obtained LODs were below these clinically relevant concentrations [61].

The sensor’s reproducibility was evaluated using independently fabricated electrodes (*n* = 3), as shown in Figure 4D. The results show consistent current responses across electrodes for PBS, 5 mM, and 10 mM glucose concentrations, indicating good fabrication reproducibility and stability of the enzyme immobilization process. The relatively low electrode-to-electrode variation suggests that the glutaraldehyde crosslinking strategy provides sufficiently consistent enzyme immobilization to support reproducible electrochemical responses [62]. Under the equimolar conditions used in this initial interference assessment, the glucose sensor showed a substantially greater response to glucose (5 mM) than to the tested non-target species (Figure 4E), consistent with GOx substrate specificity. These measurements were intended to assess analytical cross-reactivity under controlled conditions rather than to reproduce the relative concentrations encountered in biological samples. The preferential response toward glucose is consistent with GOx’s enzymatic specificity for glucose oxidation, while the selected operating potential and electrode interface may also reduce electrochemical contributions from species such as AA and UA. Future studies using physiologically representative interferent concentrations will therefore be required to establish selectivity under clinically relevant conditions [55].

### 3.4. Analytical Performance of the Adiponectin and Leptin Immunosensors

**Figure 5:**
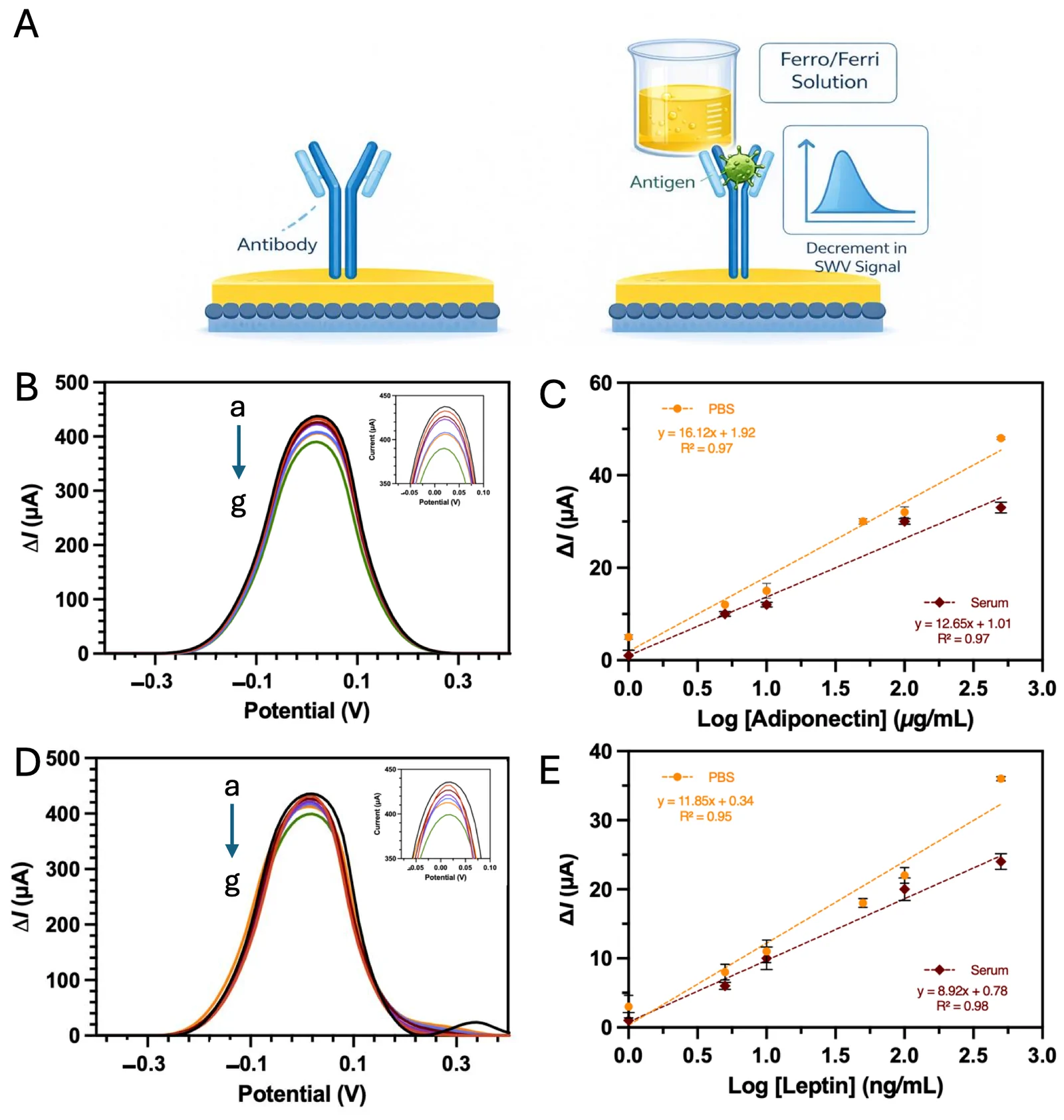
Electrochemical detection of leptin and adiponectin using immunosensors. (**A**) Schematic illustration of antibody-based antigen detection using a ferro/ferricyanide redox probe, where target binding decreases the SWV response. (**B**) SWV responses obtained at increasing adiponectin concentrations: (a) 0, (b) 1, (c) 5, (d) 10, (e) 50, (f) 100, and (g) 500 µg mL^−1^ in PBS and (**C**) calibration plots for adiponectin detection in PBS and human serum. (**D**) SWV responses obtained at increasing leptin concentrations: (a) 0, (b) 1, (c) 5, (d) 10, (e) 50, (f) 100, and (g) 500 ng mL^−1^ in PBS and the (**E**) calibration plots for leptin detection in PBS and human serum.

Figure 5A illustrates the sensing principle and SWV responses of the antibody-functionalized LSGE/Au platform toward adiponectin and leptin. After antigen binding, the antibody–antigen complex restricts access of the ferri/ferrocyanide redox probe to the electrode surface, resulting in a concentration-dependent decrease in peak current [27]. This behavior is consistent with our previous immunosensor, in which increasing biomolecular coverage reduced interfacial electron transfer and consequently decreased the ferri/ferrocyanide current [27]. In the present design, an additional insulation step was introduced to more precisely define and maintain an identical exposed area for each working electrode. This modification aimed to minimize variation in the electrochemically accessible area during Au deposition, biofunctionalization, and subsequent electrochemical measurements.

The resulting calibration curves show comparable responses for adiponectin (Figure 5B,C) and leptin (Figure 5D,E). Adiponectin exhibited sensitivities of 16.12 and 12.65 µA per log increase in concentration in PBS and serum, respectively, whereas leptin showed sensitivities of 11.85 and 8.92 µA per log increase in concentration. Their LODs were estimated at 2.55 and 3.36 µg/mL for adiponectin and 2.86 and 5.23 ng/mL for leptin in PBS and analyte-spiked 10% human serum, respectively. The investigated concentration ranges for adiponectin (1–500 µg/mL) and leptin (1–500 ng/mL) encompass reported circulating concentrations in adults. Serum adiponectin concentrations in healthy populations typically range from several to tens of µg/mL, whereas leptin concentrations strongly depend on sex and body mass index, with reported reference intervals of 0.33–19.85 ng/mL in men and 3.60–54.86 ng/mL in women [63,64]. The obtained LODs therefore fall within or near the lower portion of these reported circulating concentration ranges, supporting the physiological relevance of the analytical ranges investigated. Another interesting finding is that the difference between the two biomarker responses is substantially smaller than that observed in our previous study, particularly for serum measurements [27]. In the earlier platform, adiponectin retained a relatively strong serum response, whereas leptin showed a pronounced reduction in sensitivity. The previous calibration curves likewise showed a much larger separation between the serum sensitivities of adiponectin and leptin [27].

The more closely matched responses may partly reflect improved geometric control introduced during electrode fabrication, in which the exposed sensing area of each WE was standardized using the Kapton-defined opening. Batch-to-batch studies of laser-induced graphene electrodes have shown that manufacturing variability can measurably affect electrochemical performance, while improved fabrication control can reduce sensor-to-sensor variation [65,66]. Although the previous multiplex design already demonstrated comparable electrochemical characteristics between the two working electrodes, including closely matched electrochemically active surface areas, variations in nanostructured electrode morphology and surface functionalization can still influence antibody loading, redox-probe accessibility, and the magnitude of the immunosensor response [27,65,66,67]. Nevertheless, this explanation should be regarded as a plausible contributing mechanism rather than a definitive cause, because biomarker-specific antibody–antigen interactions, differences in surface coverage, and matrix-dependent effects in serum can also influence electrochemical immunosensor responses [67].

In addition, although the antibody-based recognition interfaces have shown target specificity in our previous work [27], interference testing under physiologically representative target-to-interferent concentration ratios was not repeated for the adiponectin and leptin channels in the present four-electrode architecture. Such experiments will be required to establish clinically relevant selectivity of these sensing interfaces within the integrated platform.

The analytical performance of the four independently functionalized sensing channels is summarized in Table 1, including the tested calibration ranges, sensitivities, coefficients of determination, and LODs obtained in PBS and analyte-spiked 10% human serum.

### 3.5. Combinatorial Analysis

**Figure 6:**
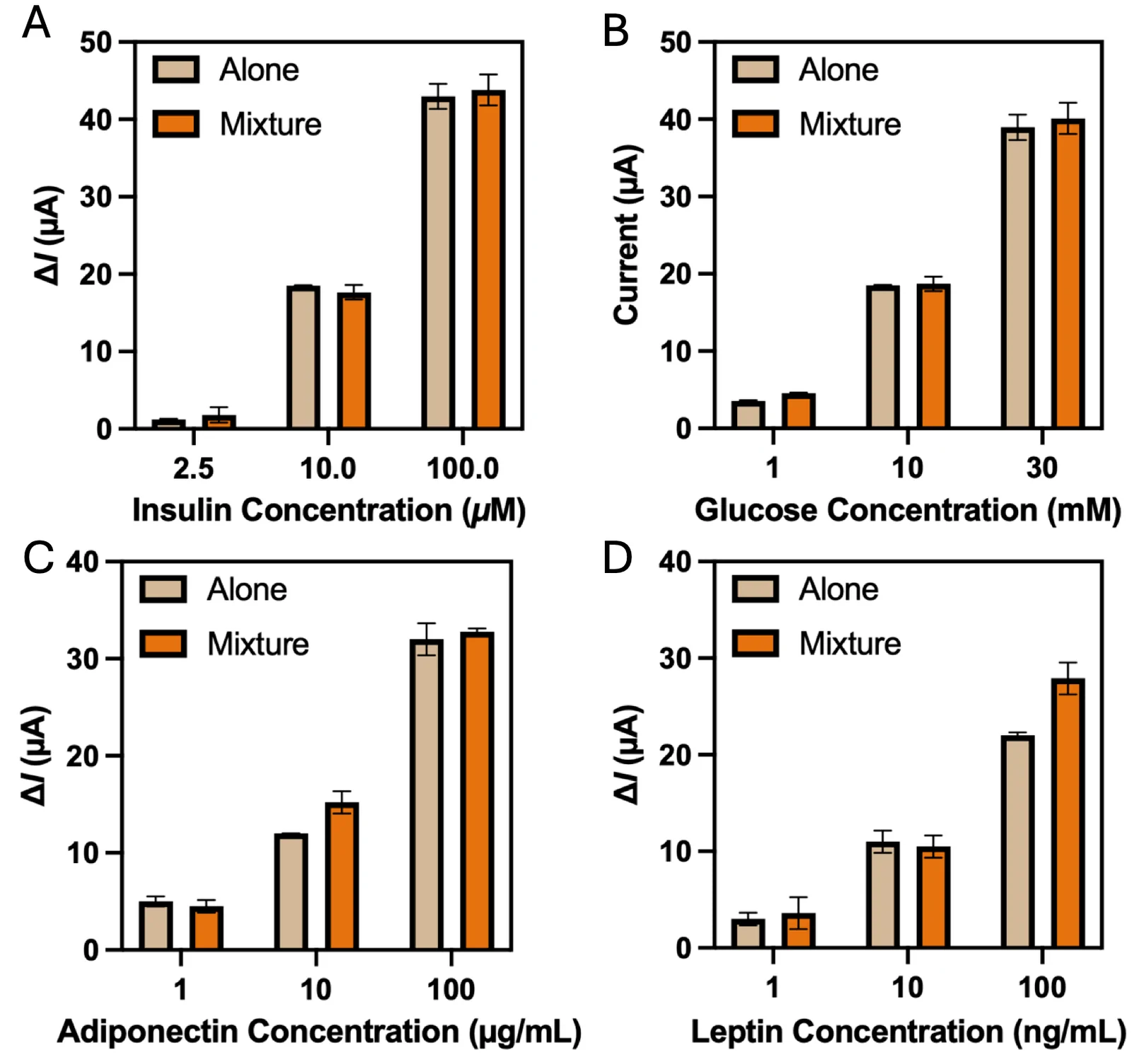
Combinatorial analysis of the four sensing channels. Comparison of the responses obtained for each target analyte measured individually and in a four-analyte mixture: (**A**) insulin at 2.5, 10, and 100 µM; (**B**) glucose at 1, 10, and 30 mM; (**C**) adiponectin at 1, 10, and 100 µg mL^−1^; and (**D**) leptin at 1, 10, and 100 ng mL^−1^. For each panel, the target analyte concentration was varied while the other three biomarkers were held at fixed concentrations.

To assess the compatibility of the four sensing interfaces under a common mixed-analyte condition, each channel was evaluated at three target concentrations in the presence of fixed concentrations of the other three biomarkers (Figure 6). This experiment was intended as a preliminary combinatorial analysis rather than a demonstration of fully simultaneous four-analyte quantification. Electrochemical sensor arrays rely on spatially distinct sensing interfaces for multi-analyte detection, but shared sample environments and differing assay requirements can still influence individual channel responses [68].

Overall, responses in mixed-analyte samples remained comparable to those for the corresponding analyte alone at many tested concentrations, although measurable deviations were observed. For insulin, the mixed-sample response was approximately 50% higher at 2.5 µM, whereas differences at 10 and 100 µM were small. For glucose, the response was approximately 28% higher at 1 mM but differed by only approximately 1–3% at 10 and 30 mM. For adiponectin, the mixed condition produced responses approximately 10% lower, 27% higher, and 3% higher at 1, 10, and 100 µg/mL, respectively. For leptin, the corresponding changes were approximately 20% higher, 5% lower, and 27% higher at 1, 10, and 100 ng/mL. These results show that all four channels remained responsive in the mixed sample, while indicating concentration-dependent changes in signal magnitude.

The observed deviations suggest that crosstalk or other mixed-sample effects cannot be excluded. Potential contributing factors include nonspecific interactions at the sensing interfaces, changes in interfacial blocking, and differences in incubation and measurement requirements among the four sensing chemistries [69,70]. Because this experiment used a common sample compartment and sequential electrochemical readout, chemical crosstalk cannot be fully distinguished from effects associated with incubation time or measurement sequence.

The sensing interfaces also require different signal-transduction conditions. The insulin aptasensor uses a tethered methylene-blue reporter and the glucose sensor incorporates a ferrocene mediator, whereas the adiponectin and leptin immunosensors require an external ferri/ferrocyanide redox probe after target incubation. The current configuration therefore enables sequential interrogation of the four channels from the same mixed sample, but not a single-step simultaneous readout. Future integration of spatially controlled reagent delivery, for example through microfluidic compartmentalization or sequential reagent routing, could help isolate the sensing chemistries and independently deliver the immunosensor redox probe [69,71,72].

Therefore, this experiment represents an early-stage demonstration of four-channel compatibility. Further optimization of channel isolation, incubation timing, and reagent delivery will be required for quantitative simultaneous detection of all four biomarkers in clinical samples.

### 3.6. Comparison with Representative Multi-Analyte Electrochemical Biosensing

As summarized in Table 2, previous multi-analyte electrochemical platforms have combined enzymatic and immunoassay formats, enzymatic and aptamer-based sensing, or multiple affinity-based interfaces. The reported concentration ranges and LODs further illustrate the diversity in analytical performance across these platforms. However, differences in sensing mechanisms, sample matrices, calibration approaches, and LOD calculation methods should be considered when interpreting these values. The contribution of the present work is therefore not the introduction of a new biorecognition chemistry, but the co-integration of four complementary insulin-resistance-associated biomarkers within a single LSG architecture, with each working electrode independently functionalized using a recognition and transduction strategy suited to its target. This design brings together GOx-mediated catalytic sensing, redox-labeled aptamer sensing, and antibody-based interfacial sensing within one platform, while simultaneously covering biomarker pairs relevant to both glucose–insulin homeostasis and adipose tissue dysfunction.

The selected biomarkers represent two complementary aspects of insulin resistance. Glucose and insulin form the biomarker pair used to calculate HOMA-IR, whereas adiponectin and leptin are used to derive the A/L ratio. HOMA-IR primarily reflects fasting glucose–insulin homeostasis, while the A/L ratio provides complementary information related to adipose tissue dysfunction and inflammatory status [6,7,9,10,11]. Integrating both biomarker pairs within the same four-working-electrode platform therefore provides a basis for assessing these related but distinct metabolic processes using a unified sensing architecture. However, calculating and clinically validating HOMA-IR and the A/L ratio remain beyond the scope of the present study. Although a preliminary mixed-analyte experiment was performed using all four biomarkers in a common sample, the sensing interfaces require different electrochemical readout conditions; the present platform employs sequential channel interrogation rather than fully simultaneous single-step detection.

## 4. Conclusions

This work demonstrates a hybrid, multi-biorecognition, laser-scribed graphene platform for electrochemical detection of four biomarkers associated with insulin resistance: glucose, insulin, adiponectin, and leptin. The platform integrates three distinct recognition strategies within a common electrode architecture, combining GOx-based enzymatic detection for glucose, a methylene-blue-labeled aptamer for insulin, and antibody-based immunosensing for adiponectin and leptin.

The four-biomarker configuration integrates complementary analytes associated with insulin resistance within a common sensing platform. Glucose and insulin correspond to the biomarker pair used in HOMA-IR, whereas adiponectin and leptin correspond to the biomarker pair used to derive the A/L ratio, thereby bringing together biomarkers associated with glucose–insulin homeostasis and adipose tissue dysfunction within a single sensing architecture.

Overall, this work demonstrates that four complementary insulin-resistance-associated biomarkers can be accommodated within a common LSG platform using independently tailored enzymatic, aptamer-based, and antibody-based sensing interfaces. Each sensing channel exhibited a concentration-dependent response when evaluated independently, and a preliminary mixed-analyte study showed that all four channels remained responsive in the presence of the other biomarkers, although some concentration-dependent deviations were observed. Because the different sensing interfaces require distinct electrochemical readout conditions, the present platform employs sequential rather than fully simultaneous interrogation. Further optimization of inter-channel isolation, reagent delivery, and analytical performance in clinically relevant samples will be required for quantitative simultaneous four-analyte detection.

## Figures and Tables

**Table 1:** Summary of the analytical performance of the individual sensing channels in PBS and analyte-spiked 10% human serum.

Biomarker	Biorecognition	Range	Calibration	Sensitivity/Slope, PBS	Sensitivity/Slope, Serum	R^2^, PBS/serum	LOD, PBS/serum
Insulin	MB-labeled aptamer/SWV	2.5–100 µM	ΔI vs. ln(C)	11.1 µA per ln unit	5.79 µA per ln unit	0.99/0.99	2.29/2.71 µM
Glucose	GOx/Fc enzymatic/chronoamperometry	1–30 mM	Current vs. C	1.29 µA mM^−1^	1.19 µA mM^−1^	0.98/0.98	0.57/1.06 mM
Adiponectin	Antibody/SWV with ferri/ferrocyanide	1–500 µg mL^−1^	ΔI vs. log_10_(C)	16.12 µA decade^−1^	12.65 µA decade^−1^	0.97/0.97	2.55/3.36 µg mL^−1^
Leptin	Antibody/SWV with ferri/ferrocyanide	1–500 ng mL^−1^	ΔI vs. log_10_(C)	11.85 µA decade^−1^	8.92 µA decade^−1^	0.95/0.98	2.86/5.23 ng mL^−1^

**Table 2:** Comparison of representative multi-analyte electrochemical biosensing platforms integrating different recognition strategies.

Study	Target Analytes	Biorecognition Strategy	Platform	Biological Matrix	Reported Concentration Range	LOD	Key Feature
Wang et al., 2003 [13]	Glucose, insulin	Enzymatic + immunoassay	Microfluidic electrochemical biochip	—	G: 0–10 mM; I: 10 pM–10 nM	G: 100 µM I: 10 pM	Early dual-assay integration for glucose and insulin
Nandhakumar et al., 2024 [24]	Glucose, insulin	GOx enzymatic + MB-labeled insulin aptamer	Two-electrode electrochemical chip	Serum and saliva	G: 2.5–15 mM I: 0.5–50 nM	G: 47 µM I: 800 pM	Enzymatic–aptamer detection with low crosstalk
Yen et al., 2024 [73]	AFP, CEA	Aptamer + aptamer	LSG electrochemical platform	PBS and serum	A: 0.75–100 ng/mL C: 0.75–100 ng/mL	A: 13.68 pg/mL C: 50.66 pg/mL	Dual-protein detection with aptamer-functionalized LSG interfaces.
Rizalputri et al., 2025 [27]	Adiponectin, leptin	Antibody + antibody	Two-electrode Au-nanostructured LSGE	Serum	Ad:1–500 µg/mL L: 1–500 ng/mL	Ad: 0.59 µg/mL L: 0.18 ng/mL	LSG immunosensing of adiponectin and leptin.
This work	Glucose, insulin, adiponectin, leptin	GOx enzymatic + MB-labeled insulin aptamer + Antibody	Four-working-electrode hybrid LSG platform; LSG/Au for insulin/adipokine sensing and bare LSG for glucose sensing	PBS and 10% serum	G: 1–30 mM I: 2.5–100 µM Ad: 1–500 µg/mL L: 1–500 ng/mL	G: 0.57 mM I: 2.29 µM Ad: 2.55 µg/mL L: 2.86 ng/mL	Three biorecognition modalities integrated across four LSG working electrodes.

G: glucose; I: insulin; A: AFP; C: CEA; Ad: adiponectin; L: leptin.

## Data Availability

Data will be made available upon request.

## Supplementary Materials

The following supporting information can be downloaded at https://www.mdpi.com/article/10.3390/bios16100545/s1, Figure S1: Optimization of the insulin aptamer-based electrochemical sensor; Figure S2: Electrochemical responses of insulin, glucose, adiponectin, and leptin sensing channels in 10% human serum spiked with increasing concentrations of the corresponding analytes.

## Abbreviations

The following abbreviations are used in this manuscript:

A/L	Adiponectin/leptin ratio
AA	Ascorbic acid
BSA	Bovine serum albumin
CE	Counter electrode
CV	Cyclic voltammetry
EDC	1-Ethyl-3-(3-dimethylaminopropyl)carbodiimide
FAD	Flavin adenine dinucleotide
FADH_2_	Reduced flavin adenine dinucleotide
Fc	Ferrocene
GA	Glutaraldehyde
GOx	Glucose oxidase
HEGC	Hyperinsulinemic-euglycemic glucose clamp
HOMA-IR	Homeostasis model assessment of insulin resistance
HPLC	High-performance liquid chromatography
IR	Insulin resistance
LOD	Limit of detection
LSG	Laser-scribed graphene
LSGE	Laser-scribed graphene electrode
MB	Methylene blue
MCH	6-Mercapto-1-hexanol
MPA	3-Mercaptopropionic acid
NHS	N-Hydroxysuccinimide
PBS	Phosphate-buffered saline
PI	Polyimide
QUICKI	Quantitative insulin sensitivity check index
RE	Reference electrode
SAM	Self-assembled monolayer
SD	Standard deviation
SEM	Scanning electron microscopy
SWV	Square-wave voltammetry
TCEP	Tris(2-carboxyethyl)phosphine
UA	Uric acid
WE	Working electrode

## References

[1] Zhao X., An X., Yang C., Sun W., Ji H., Lian F. (2023). The Crucial Role and Mechanism of Insulin Resistance in Metabolic Disease. Front. Endocrinol..

[2] Yu L., Qian J., Li X., Tian M., Bai X., Yang J., Deng R., Lu C., He X., Lu A. (2026). Insulin Resistance: Mechanisms and Therapeutic Interventions. Mol. Biomed..

[3] Accili D., Deng Z., Liu Q. (2025). Insulin Resistance in Type 2 Diabetes Mellitus. Nat. Rev. Endocrinol..

[4] Parcha V., Heindl B., Kalra R., Li P., Gower B., Arora G., Arora P. (2022). Insulin Resistance and Cardiometabolic Risk Profile among Nondiabetic American Young Adults: Insights from NHANES. J. Clin. Endocrinol. Metab..

[5] Matthews D.R., Hosker J.R., Rudenski A.S., Naylor B.A., Treacher D.F., Turner R.C. (1985). Homeostasis Model Assessment: Insulin Resistance and β-Cell Function from Fasting Plasma Glucose and Insulin Concentrations in Man. Diabetologia.

[6] Frühbeck G., Catalán V., Rodríguez A., Gómez-Ambrosi J. (2018). Adiponectin-Leptin Ratio: A Promising Index to Estimate Adipose Tissue Dysfunction. Relation with Obesity-Associated Cardiometabolic Risk. Adipocyte.

[7] Groeneveld M.P., Huang-Doran I., Semple R.K. (2012). Adiponectin and Leptin in Human Severe Insulin Resistance—Diagnostic Utility and Biological Insights. Biochimie.

[8] Castela I., Morais J., Barreiros-Mota I., Silvestre M.P., Marques C., Rodrigues C., Ismael S., Araújo J.R., Ângelo-Dias M., Martins C. (2023). Decreased Adiponectin/Leptin Ratio Relates to Insulin Resistance in Adults with Obesity. Am. J. Physiol. Endocrinol. Metab..

[9] Zaletel J., Pongrac Barlovic D., Prezelj J. (2010). Adiponectin-Leptin Ratio: A Useful Estimate of Insulin Resistance in Patients with Type 2 Diabetes. J. Endocrinol. Investig..

[10] Inoue M., Yano M., Yamakado M., Maehata E., Suzuki S. (2006). Relationship between the Adiponectin-Leptin Ratio and Parameters of Insulin Resistance in Subjects without Hyperglycemia. Metabolism.

[11] Jung C.-H., Rhee E.-J., Choi J.-H., Bae J.-C., Yoo S.-H., Kim W.-J., Park C.-Y., Mok J.O., Kim C.H., Lee W.-Y. (2010). The Relationship of Adiponectin/Leptin Ratio with Homeostasis Model Assessment Insulin Resistance Index and Metabolic Syndrome in Apparently Healthy Korean Male Adults. Korean Diabetes J..

[12] Fiedorova K., Augustynek M., Kubicek J., Kudrna P., Bibbo D. (2022). Review of Present Method of Glucose from Human Blood and Body Fluids Assessment. Biosens. Bioelectron..

[13] Wang J., Ibáñez A., Chatrathi M.P. (2003). On-Chip Integration of Enzyme and Immunoassays: Simultaneous Measurements of Insulin and Glucose. J. Am. Chem. Soc..

[14] Martin R.M., Patel R., Oken E., Thompson J., Zinovik A., Kramer M.S., Vilchuck K., Bogdanovich N., Sergeichick N., Foo Y. (2013). Filter Paper Blood Spot Enzyme Linked Immunoassay for Adiponectin and Application in the Evaluation of Determinants of Child Insulin Sensitivity. PLoS ONE.

[15] Beduk T., Beduk D., Hasan M.R., Guler Celik E., Kosel J., Narang J., Salama K.N., Timur S. (2022). Smartphone-Based Multiplexed Biosensing Tools for Health Monitoring. Biosensors.

[16] Fu Q., Schoenhoff F.S., Savage W.J., Zhang P., van Eyk J.E. (2010). Multiplex Assays for Biomarker Research and Clinical Application: Translational Science Coming of Age. Proteom. Clin. Appl..

[17] da Silva E.T.S.G., Souto D.E.P., Barragan J.T.C., de F. Giarola J., de Moraes A.C.M., Kubota L.T. (2017). Electrochemical Biosensors in Point-of-Care Devices: Recent Advances and Future Trends. ChemElectroChem.

[18] Kim J., Jeong J., Ko S.H. (2024). Electrochemical Biosensors for Point-of-Care Testing. Biodes. Manuf..

[19] Nam K.H., Abdulhafez M., Castagnola E., Tomaraei G.N., Cui X.T., Bedewy M. (2022). Laser Direct Write of Heteroatom-Doped Graphene on Molecularly Controlled Polyimides for Electrochemical Biosensors with Nanomolar Sensitivity. Carbon.

[20] Lin J., Peng Z., Liu Y., Ruiz-Zepeda F., Ye R., Samuel E.L.G., Yacaman M.J., Yakobson B.I., Tour J.M. (2014). Laser-Induced Porous Graphene Films from Commercial Polymers. Nat. Commun..

[21] Thaweeskulchai T., Sakdaphetsiri K., Schulte A. (2024). Ten Years of Laser-Induced Graphene: Impact and Future Prospect on Biomedical, Healthcare, and Wearable Technology. Microchim. Acta.

[22] Behrent A., Borggraefe V., Baeumner A.J. (2024). Laser-Induced Graphene Trending in Biosensors: Understanding Electrode Shelf-Life of This Highly Porous Material. Anal. Bioanal. Chem..

[23] Liu M., Zhao G., Yang C., Wang H., Cheng J. (2023). An Enzymatic Electrochemical Biosensing Interface Developed by The Laser-Induced Graphene Electrode. Adv. Mater. Interfaces.

[24] Nandhakumar P., Djassemi O., Raucci A., Chang A.Y., Cheung C., Dugas Y., Silberman J., Morales-Fermin S., S Sandhu S., Reynoso M. (2024). Simultaneous and Rapid Detection of Glucose and Insulin: Coupling Enzymatic and Aptamer-Based Assays. Anal. Chem..

[25] Wu Y., Midinov B., White R.J. (2019). Electrochemical Aptamer-Based Sensor for Real-Time Monitoring of Insulin. ACS Sens..

[26] Schoukroun-Barnes L.R., Macazo F.C., Gutierrez B., Lottermoser J., Liu J., White R.J. (2016). Reagentless, Structure-Switching, Electrochemical Aptamer-Based Sensors. Annu. Rev. Anal. Chem..

[27] Rizalputri L.N., de Oliveira Filho J.I., Shetty S.S., le Roux E., Bukhamsin A., Alshehri B., Mani V., Salama K.N. (2025). A Multiplexed Laser-Scribed Graphene Immunosensor for Simultaneous Detection of Adiponectin and Leptin: A Point-of-Care Solution for Early Insulin Resistance Diagnosis. Sens. Actuators Rep..

[28] Arshavsky-Graham S., Heuer C., Jiang X., Segal E. (2022). Aptasensors versus Immunosensors—Which Will Prevail?. Eng. Life Sci..

[29] Beduk T., Ait Lahcen A., Tashkandi N., Salama K.N. (2020). One-Step Electrosynthesized Molecularly Imprinted Polymer on Laser Scribed Graphene Bisphenol a Sensor. Sens. Actuators B Chem..

[30] Beduk T., Beduk D., de Oliveira Filho J.I., Zihnioglu F., Cicek C., Sertoz R., Arda B., Goksel T., Turhan K., Salama K.N. (2021). Rapid Point-of-Care COVID-19 Diagnosis with a Gold-Nanoarchitecture-Assisted Laser-Scribed Graphene Biosensor. Anal. Chem..

[31] Yoshida W., Mochizuki E., Takase M., Hasegawa H., Morita Y., Yamazaki H., Sode K., Ikebukuro K. (2009). Selection of DNA Aptamers against Insulin and Construction of an Aptameric Enzyme Subunit for Insulin Sensing. Biosens. Bioelectron..

[32] Currie L.A. (1999). Nomenclature in Evaluation of Analytical Methods Including Detection and Quantification Capabilities (IUPAC Recommendations 1995). Anal. Chim. Acta.

[33] International Council for Harmonisation of Technical Requirements for Pharmaceuticals for Human Use (ICH) (2023). Q2(R2) Validation of Analytical Procedures.

[34] Rauf S., Lahcen A.A., Aljedaibi A., Beduk T., Ilton de Oliveira Filho J., Salama K.N. (2021). Gold Nanostructured Laser-Scribed Graphene: A New Electrochemical Biosensing Platform for Potential Point-of-Care Testing of Disease Biomarkers. Biosens. Bioelectron..

[35] Shetty S.S., Rizalputri L.N., Trachioti M.G., Yuvaraja S., Mani V., Prodromidis M.I., Salama K.N. (2024). Non-Destructive Integration of Spark-Discharge Produced Gold Nanoparticles onto Laser-Scribed Graphene Electrodes for Advanced Electrochemical Sensing Applications. Surf. Interfaces.

[36] Ferreira D.C., Shetty S.S., Rizalputri L.N., Mani V., Salama K.N. (2024). Activated and Shorter Laser-Scribed Graphene Electrodes: Excellent Electrochemical Signal Amplification for Detecting Biomarkers. Microchem. J..

[37] Ferrari A.C. (2007). Raman Spectroscopy of Graphene and Graphite: Disorder, Electron-Phonon Coupling, Doping and Nonadiabatic Effects. Solid State Commun..

[38] Oberhaus F.V., Frense D., Beckmann D. (2020). Immobilization Techniques for Aptamers on Gold Electrodes for the Electrochemical Detection of Proteins: A Review. Biosensors.

[39] Weber C.J., Strom N.E., Simoska O. (2024). Electrochemical Deposition of Gold Nanoparticles on Carbon Ultramicroelectrode Arrays. Nanoscale.

[40] Dauphin-Ducharme P., Plaxco K.W. (2016). Maximizing the Signal Gain of Electrochemical-DNA Sensors. Anal. Chem..

[41] Uzawa T., Cheng R.R., White R.J., Makarov D.E., Plaxco K.W. (2010). A Mechanistic Study of Electron Transfer from the Distal Termini of Electrode-Bound, Single-Stranded DNAs. J. Am. Chem. Soc..

[42] Ricci F., Lai R.Y., Heeger A.J., Plaxco K.W., Sumner J.J. (2007). Effect of Molecular Crowding on the Response of an Electrochemical DNA Sensor. Langmuir.

[43] Shaver A., Mahlum J.D., Scida K., Johnston M.L., Aller Pellitero M., Wu Y., Carr G.V., Arroyo-Currás N. (2022). Optimization of Vancomycin Aptamer Sequence Length Increases the Sensitivity of Electrochemical, Aptamer-Based Sensors In Vivo. ACS Sens..

[44] Ferrer Pascual L., Gustafsson E., Siitonen J., Durairaj V., Laurila T. (2024). Challenges in Aptamer-Based Sensor Development Using Carbon Nanotube Networks. Sens. Diagn..

[45] McCann B., Tipper B., Shahbeigi S., Soleimani M., Jabbari M., Nasr Esfahani M. (2025). A Review on Perception of Binding Kinetics in Affinity Biosensors: Challenges and Opportunities. ACS Omega.

[46] Zhou L., Zhu R., Figueroa-Miranda G., Neis M., Offenhäusser A., Mayer D. (2024). Ratiometric Electrochemical Aptasensor with Strand Displacement for Insulin Detection in Blood Samples. Anal. Chim. Acta.

[47] Ferapontova E.E., Gothelf K.V. (2009). Effect of Serum on an RNA Aptamer-Based Electrochemical Sensor for Theophylline. Langmuir.

[48] Grabowska I., Sharma N., Vasilescu A., Iancu M., Badea G., Boukherroub R., Ogale S., Szunerits S. (2018). Electrochemical Aptamer-Based Biosensors for the Detection of Cardiac Biomarkers. ACS Omega.

[49] Kruk T., Bzowska M., Hinz A., Szuwarzyński M., Szczepanowicz K. (2021). Control of Specific/Nonspecific Protein Adsorption: Functionalization of Polyelectrolyte Multilayer Films as a Potential Coating for Biosensors. Materials.

[50] Delacruz L., Adamczak L.J., Astapova O. (2025). Postprandial Hypoglycemia Associated With Insulin Receptor Gene Variant G1146R. JCEM Case Rep..

[51] White R.J., Phares N., Lubin A.A., Xiao Y., Plaxco K.W. (2008). Optimization of Electrochemical Aptamer-Based Sensors via Optimization of Probe Packing Density and Surface Chemistry. Langmuir.

[52] Wu Y., Plaxco K.W. (2025). On the Design, Fabrication, and Operation of Electrochemical Aptamer-Based Sensors. ACS Sens..

[53] Yang J., Zhang Y. (2025). Research Progress on Aptamer Electrochemical Biosensors Based on Signal Amplification Strategy. Sensors.

[54] He L., Huang R., Xiao P., Liu Y., Jin L., Liu H., Li S., Deng Y., Chen Z., Li Z. (2021). Current Signal Amplification Strategies in Aptamer-Based Electrochemical Biosensor: A Review. Chin. Chem. Lett..

[55] Masson J.F. (2020). Consideration of Sample Matrix Effects and “Biological” Noise in Optimizing the Limit of Detection of Biosensors. ACS Sens..

[56] Noda T., Wanibuchi M., Kitazumi Y., Tsujimura S., Osamu S., Yamamoto M., Kano K. (2013). Diffusion-Controlled Detection of Glucose with Microelectrodes in Mediated Bioelectrocatalytic Oxidation. Anal. Sci..

[57] Phasuksom K., Sirivat A. (2022). Chronoampermetric Detection of Enzymatic Glucose Sensor Based on Doped Polyindole/MWCNT Composites Modified onto Screen-Printed Carbon Electrode as Portable Sensing Device for Diabetes. RSC Adv..

[58] Cass A.E.G., Davis G., Francis G.D., Allen H., Hill O., Aston W.J., Higgins I.J., Plotkin E.V., Scott L.D.L., Turner A.P.F. (1984). Ferrocene-Mediated Enzyme Electrode for Amperometric Determination of Glucose. Anal. Chem..

[59] Downs A.M., Plaxco K.W. (2022). Real-Time, in Vivo Molecular Monitoring Using Electrochemical Aptamer Based Sensors: Opportunities and Challenges. ACS Sens..

[60] Pham J.D., Fetter L.C., Gerson J., Kippin T.E., Plaxco K.W., Leung K.K. (2025). On the Blood Components Contributing to the Drift of Electrochemical Aptamer-Based Biosensors. ACS Sens..

[61] American Diabetes Association Professional Practice Committee for Diabetes (2026). 2. Diagnosis and Classification of Diabetes: Standards of Care in Diabetes-2026. Diabetes Care.

[62] López-Gallego F., Betancor L., Mateo C., Hidalgo A., Alonso-Morales N., Dellamora-Ortiz G., Guisán J.M., Fernández-Lafuente R. (2005). Enzyme Stabilization by Glutaraldehyde Crosslinking of Adsorbed Proteins on Aminated Supports. J. Biotechnol..

[63] Flechtner-Mors M., George S.N., Oeztuerk S., Haenle M.M., Koenig W., Imhof A., Boehm B.O., Graeter T., Mason R.A., Kratzer W. (2014). Association of Adiponectin with Hepatic Steatosis: A Study of 1,349 Subjects in a Random Population Sample. BMC Res. Notes.

[64] Cheng J., Luo Y., Li Y., Zhang F., Zhang X., Zhou X., Ji L. (2022). Sex- and Body Mass Index-Specific Reference Intervals for Serum Leptin: A Population Based Study in China. Nutr. Metab..

[65] Tang Y., Moreira G.A., Vanegas D., Datta S.P.A., McLamore E.S. (2024). Batch-to-Batch Variation in Laser-Inscribed Graphene (LIG) Electrodes for Electrochemical Sensing. Micromachines.

[66] Qian H., Moreira G., Vanegas D., Tang Y., Pola C., Gomes C., McLamore E., Bliznyuk N. (2024). Improving High Throughput Manufacture of Laser-Inscribed Graphene Electrodes via Hierarchical Clustering. Sci. Rep..

[67] Chen H., Zhang J., Huang R., Wang D., Deng D., Zhang Q., Luo L. (2023). The Applications of Electrochemical Immunosensors in the Detection of Disease Biomarkers: A Review. Molecules.

[68] Sen D., Lazenby R.A. (2024). Electrochemical Biosensor Arrays for Multiple Analyte Detection. Anal. Sens..

[69] Glatz R.T., Ates H.C., Mohsenin H., Weber W., Dincer C. (2022). Designing Electrochemical Microfluidic Multiplexed Biosensors for On-Site Applications. Anal. Bioanal. Chem..

[70] Russo M.J., Han M., Desroches P.E., Manasa C.S., Dennaoui J., Quigley A.F., Kapsa R.M.I., Moulton S.E., Guijt R.M., Greene G.W. (2021). Antifouling Strategies for Electrochemical Biosensing: Mechanisms and Performance toward Point of Care Based Diagnostic Applications. ACS Sens..

[71] Gil Rosa B., Akingbade O.E., Guo X., Gonzalez-Macia L., Crone M.A., Cameron L.P., Freemont P., Choy K.L., Güder F., Yeatman E. (2022). Multiplexed Immunosensors for Point-of-Care Diagnostic Applications. Biosens. Bioelectron..

[72] Schmidt-Speicher L.M., Länge K. (2021). Microfluidic Integration for Electrochemical Biosensor Applications. Curr. Opin. Electrochem..

[73] Yen Y.K., Huang G.W., Shanmugam R. (2024). Laser-Scribing Graphene-Based Electrochemical Biosensing Devices for Simultaneous Detection of Multiple Cancer Biomarkers. Talanta.

