# Supplementary material for: Hybrid Multi-Biorecognition Laser-Scribed Graphene Platform for Electrochemical Detection of Insulin Resistance Biomarkers
Source: Biosensors (Basel). 2026 Sep 29;16(10):545. doi: 10.3390/bios16100545 (PMC13702624; doi:10.3390/bios16100545)
Supplement: Supplementary file 1 [file biosensors-16-00545-s001.zip › biosensors-4543246-supplementary.pdf]

biosensors

MDPI

Article

Hybrid Multi-Biorecognition Laser-Scribed Graphene Platform for Electrochemical Detection of Insulin Resistance Biomarkers

Lavita Nuraviana Rizalputri [1], Saptami Suresh Shetty [1], Mario Soto Martinez [1], Eckaard Le Roux [2], Veerappan Mani [3] and Khaled Nabil Salama [1,2,*]

[1] Bioengineering Program, Biomedical Science Division, King Abdullah University of Science and Technology, Thuwal, Saudi Arabia; lavita.rizalputri@kaust.edu.sa (L.N.R.); saptami.shetty@kaust.edu.sa (S.S.S.); mario.sotomartinez@kaust.edu.sa (M.S.M.)
[2] Electrical and Computer Engineering Program, Computer, Electrical and Mathematical Science and Engineering Division, King Abdullah University of Science and Technology, Thuwal, Saudi Arabia; eckaard.leroux@kaust.edu.sa
[3] Centre for Advanced Measurement Research & Health Translation, Department of Pure and Applied Chemistry, University of Strathclyde, Glasgow G1 1XL, UK; veerappan.mani@strath.ac.uk
* Correspondence: khaled.salama@kaust.edu.sa

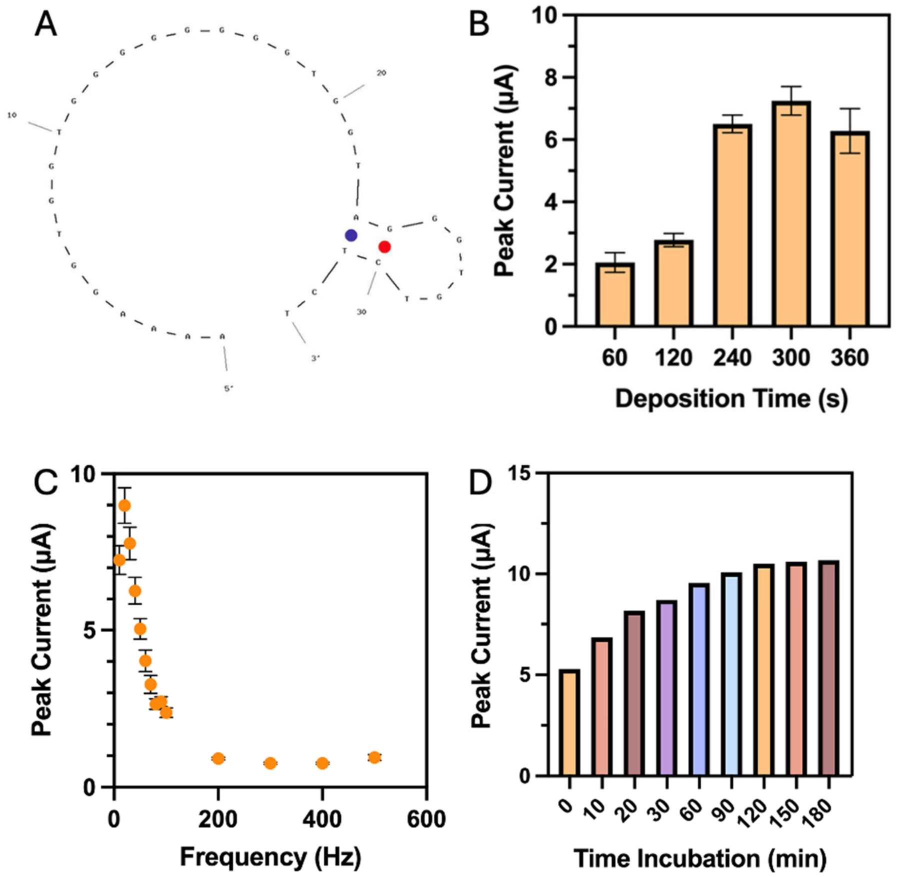

Figure S1. Optimization of the insulin aptamer-based electrochemical sensor. (A) Predicted secondary structure of the insulin aptamer generated using UNAFold. (B) Optimization of Au deposition time to improve aptamer immobilization on the Au-decorated laser-scribed graphene electrode. (C) Optimization of SWV frequency to obtain the MB electrochemical response from the immobilized aptamer. (D) Effect of post-fabrication equilibration time in PBS on the MB peak current of the aptamer-functionalized electrode.

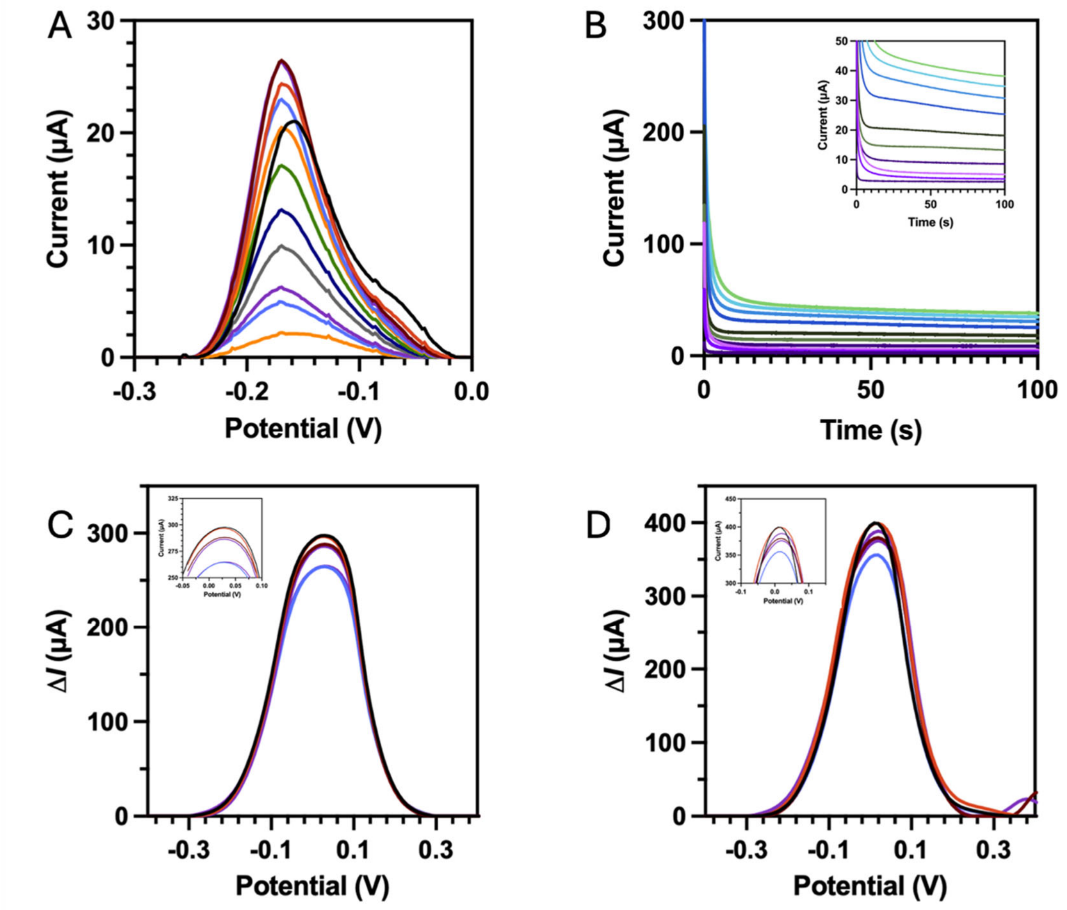

Figure S2. Electrochemical responses of insulin, glucose, adiponectin, and leptin sensing channels in 10% human serum spiked with increasing concentrations of the corresponding analytes. Figure S2. Electrochemical responses of the individual sensing channels measured in 10% human serum spiked with different concentrations of the corresponding analytes. (A) Square-wave voltammetry (SWV) responses of the insulin aptasensor at increasing insulin concentrations. (B) Chronoamperometric responses of the GOx-based glucose sensor at increasing glucose concentrations; the inset shows an enlarged view of the current–time profiles. (C) SWV responses of the adiponectin immunosensor at increasing adiponectin concentrations; the inset highlights the changes near the peak-current region. (D) SWV responses of the leptin immunosensor at increasing leptin concentrations; the inset shows the corresponding peak-current changes. All measurements were performed using 10% human serum as the sample matrix.
